# Finding a ‘new normal’ following acute illness: A qualitative study of influences on frail older people’s care preferences

**DOI:** 10.1177/0269216318817706

**Published:** 2018-12-07

**Authors:** Simon Noah Etkind, Natasha Lovell, Caroline Jane Nicholson, Irene J Higginson, Fliss EM Murtagh

**Affiliations:** 1 Cicely Saunders Institute, Florence Nightingale Faculty of Nursing, Midwifery & Palliative Care, King’s College London, London, UK; 2 St Christopher’s Hospice, London, UK; 3 King’s College Hospital NHS Foundation Trust, London, UK; 4 Wolfson Palliative Care Research Centre, Hull York Medical School, University of Hull, Hull, UK

**Keywords:** Preferences, aged, frailty, qualitative research, hospitalisation

## Abstract

**Background:**

The frail older population is growing, and many frail older people have episodes of acute illness. Patient preferences are increasingly considered important in the delivery of person-centred care and may change following acute illness.

**Aim:**

To explore influences on the care preferences of frail older people with recent acute illness.

**Design:**

Qualitative in-depth individual interviews, with thematic analysis.

**Setting/participants:**

Maximum variation sample of 18 patients and 7 nominated family carers from a prospective cohort study of people aged over 65, scoring ⩾5 on the Clinical Frailty Scale, and with recent acute illness, who were not receiving specialist palliative care. Median patient age was 84 (inter-quartile range 81–87), 53% female. Median frailty score 6 (inter-quartile range 5–7).

**Results:**

Key influences on preferences were illness and care context, particularly hospital care; adaptation to changing health; achieving normality and social context. Participants focused on the outcomes of their care; hence, whether care was likely to help them ‘get back to normal’, or alternatively ‘find a new normal’ influenced preferences. For some, acute illness inhibited preference formation. Participants’ social context and the people available to provide support influenced place of care preferences. We combined these findings to model influences on preferences.

**Conclusion:**

‘Getting back to normal’ or ‘finding a new normal’ are key focuses for frail older people when considering their preferences. Following acute illness, clinicians should discuss preferences and care planning in terms of an achievable normal, and carefully consider the social context. Longitudinal research is needed to explore the influences on preferences over time.

**What is already known about the topic?**

- To be person-centred, care should take into account individual preferences.
- Some influences on care preferences in older people have been described. These include the family and care context, individual response and illness-related factors.
- Older people living with frailty are at high risk of acute illness episodes; the influences on preferences in the context of frailty and recent acute illness have not been explored.

**What this paper adds?**

- Achieving normality, by ‘getting back to normal’ or ‘finding a new normal’ influences preferences in frail older people with recent acute illness, as participants seek care that will help them find this normality.
- Preferences are also influenced by the way people respond to changing health and care experiences.
- We propose a model of influences on care preferences in the context of recent acute illness.

**Implications for practice, theory or policy**

- The influences described in this model can act as a guide for discussion and elicitation of current and future care preferences in this population.
- When addressing care preferences with patients and families, clinicians should discuss what may be an achievable normal for them within their social context.
- Prospective longitudinal study will allow exploration of influences on the *stability* of care preferences following acute illness.

## Background

Care centred on the needs and preferences of the individual – person-centred care – is widely accepted as gold standard,^1,2^ especially for older people living with syndromes such as multimorbidity and frailty.^3,4^ Person-centred care should take into account patients’ preferences, and so delivery of such care is contingent on a good understanding of care preferences.^5–7^ ‘Achievement of preferences’ is an accepted quality indicator in palliative care.^8^

There is no consensus definition of a care preference, but one useful designation is that they are statements of what individuals want from their care,^9,10^ including aspects such as the context in which care is delivered, degree of involvement in care and decisions, care relationships and care outcomes.^5,11^ In psychology, a preference is based on cognitive evaluation of the value one puts on something, and the likelihood of it occurring.^10,12^ However, from a sociological perspective, preferences are more than cognitive constructs. Research indicates that affective factors,^13,14^ and family and care context,^15^ are vital influences on preferences in the older population. Healthcare decisions in older people are made within a ‘decision ecology’.^16^ An ecological approach to preferences in this population is therefore most appropriate.^9,16,17^

Taking this approach means accepting that people’s preferences are neither static, nor independent of their social world; rather preferences are shaped and influenced by a wide range of illness, individual and social factors.^15^ These influences may mean that preferences are labile. If we are to anticipate how preferences may evolve, and thereby deliver care that is responsive to preferences over the course of an illness trajectory, it is important to go beyond preferences themselves^18–20^ and understand the personal and social influences that underlie preferences.

The influences on preferences have been described in some older populations,^15^ but we do not know how preferences are influenced in the context of recent acute illness, defined here operationally as unexpected illness requiring hospitalisation or urgent assessment by services beyond primary care.^21^ Acute illness requiring hospitalisation is frequent in older populations and may be a catalyst for change in preferences^22^; hence, it is important to investigate influences on preferences following acute illness.

It is particularly important to understand this in the growing population of frail older people, because frailty, representing a syndrome of loss of functional reserve with increased vulnerability to adverse outcomes and loss of function,^23^ is a risk factor for acute illness and hospitalisation,^4,24,25^ with poor associated outcomes.^26^ To deliver responsive, person-centred care in this population, we need to know how preferences are influenced following acute illness. This study therefore aims to explore the influences on care preferences in frail older people with recent acute illness.

## Methods

### Design and theoretical underpinning

In this qualitative study, participants undertook single in-depth semi-structured interviews. We explored preferences from an ecological systems perspective, which recognises the importance of the contexts within which preferences are developed.^9,16^ To explore influences on preferences in more detail, we used the framework provided by response shift theory.^22^ Response shift describes how a *catalyst* (change in health status) may change a person’s internal evaluation of an *outcome* (quality of life), by means of a series of *mechanisms*, and in the presence of *antecedents* (individual and social context). It proposes to explain why objective changes in health status do not necessarily result in equivalent changes to quality of life. Response shift may occur through reprioritization (i.e. changes in importance of competing priorities), or through reconceptualization (i.e. changes in how a priority or underlying construct is conceptualised or understood). Both of these may reflect a change or influence on preferences.^22,27^ We selected response shift theory because it helps to understand how and when change occurs during adaptation to serious illness,^22^ without specifying whether that change will be recovery (as in crisis theory of illness),^28^ or deterioration (as in reintegration of loss theory).^29^ This is important in a population who can be considered to be at a liminal stage.^30^ Response shift has previously been used to investigate preferences in seriously ill populations.^31^

### Setting and selection of participants

Older people and their nominated informal carers were purposively sampled from the population of an ongoing mixed-methods prospective cohort study of frail older people with recent hospitalisation (the International Access, Rights and Empowerment Study II (IARE II)).^32^ IARE II took place in two acute hospitals, one sub-acute hospital, and one acute community service in London, UK. Criteria for inclusion were age ⩾65, Rockwood Clinical Frailty Score ⩾5 - corresponding to ‘more evident slowing, requiring help with higher order activities of daily living’,^33^ and an illness requiring hospital admission or two acute care service contacts in the last 6 months. Exclusion criteria were patients receiving specialist palliative care, and those lacking capacity with no personal consultee; 90 patients and 31 nominated carers participated in the IARE II study (351 were screened and 192 eligible). We sought a maximum-variation sample of IARE II participants to enable exploration of preferences in a diverse group.^34^ Participants were assessed sequentially against the following sampling criteria (see also supplementary material A):

- Age (65–85 vs >85);
- Hospital admissions in the last 6 months (>one vs ⩽one);
- Functional status (Australian-modified Karnofsky Performance Status (AKPS) >50 vs ⩽50);
- Living status (living alone vs living with someone).

We chose these criteria because they may influence preferences in older populations.^15^ Sampling continued alongside analysis until no new themes relating to the study question were identified during analysis, representing thematic saturation.^34^

### Data collection

All participants were informed when they provided written or witnessed consent, of the possibility of a qualitative interview. Participants and carers (where nominated) were approached by telephone or in-person to arrange an interview. They were informed the interview would explore illness experiences, care preferences and priorities. We considered individual interviews most appropriate to explore what can be sensitive topics. Participants could decline interviews, though none did. Interviews were undertaken between February 2017 and February 2018 in a place of the participants choosing. This was usually their own home, but some were conducted in hospital. Older people chose whether to be interviewed alone or with their nominated carer. If alone, nominated carers were interviewed separately. If a potential participant lacked capacity to consent, their nominated carer was approached as a proxy.^35^

The topic-guide is summarised in Table 1. A carer topic-guide was followed for carer interviews, focusing on carer experience of patient illness, and their views of the linked participant’s preferences. For joint interviews, the patient topic-guide was followed, but opportunity was given to the carer to express their views. Questions about illness experience were included, based on evidence that experience influences preferences.^15,36^ We used a distress protocol where necessary. All interviews were conducted by one male researcher (S.N.E.) with experience and training in in-depth interviewing. S.N.E. has a medical background, and we considered how this affected the data using post-interview field notes, a reflexive diary, and discussion within the research team.

**Table 1.** Summary of topic-guide (see supplementary material B for full patient and carer topic-guides).

Experience of illness • Recent and longer term illness context • Main limitations on health
Experience of care • Experience in each recent care setting
Care preferences • How do you make choices about your health • What is important with regards your health and care • How do you decide what is important • Does this change over time, what makes it change
Ideas about the future • How do you see your health changing • Future care preferences

### Analysis

Interviews were analysed thematically using NVIVO version 10 (QSR International (UK) Ltd).^34^ Interviews were recorded, transcribed verbatim and anonymised before analysis. Transcripts were read and re-read, and coded inductively for themes relevant to experience of illness and influences on preferences. A coding frame was developed (by S.N.E.), and checked against the data to ensure fit. Three transcripts were double-coded by another researcher (N.L.), who independently produced a coding frame which was triangulated with the main framework. The coding framework was reviewed and related codes were grouped into themes. During coding and theme generation, we were mindful of the theoretical underpinning in this area. Specifically, we sought to understand how our data related to the antecedents, mechanisms and catalysts proposed by response shift theory. We then interpreted our thematic model in the context of relevant literature classifying influences on preferences to assess fit with existing knowledge, paying attention to divergence^15,36^ (see supplementary material C for further details).

### Patient and public involvement

Patient, carer and public involvement (PPI) representatives formed a project advisory group for this study. They contributed via face to face and email communication to development of the topic-guide, study materials and interpretation of emergent findings in order to heighten relevance to patients.^37^ Participants themselves did not review transcripts or findings.

### Ethical approval

This study received ethical approval from the UK Health Research Authority (reference 16/LO/2048).

## Results

Eighteen participants were sampled for interviews, nominating seven participating caregivers (see Table 2 for participant details). One participant could not complete an interview due to cognitive impairment; hence, the carer was interviewed. One participant could only give a partial interview. In all but one case, patients with carers chose joint interviews. Interviews lasted median 35 min (range 23–66 min).

**Table 2.** Characteristics of participants.

Characteristic	Participants (*n* = 18)	
Age	Median 84	Inter-quartile range (IQR) 81–87 range 70–93
Clinical Frailty Scale (CFS)^a^	Median 6	IQR 5–7; range 5–8
Australian-modified Karnofsky Performance Status (AKPS)^b^	Median 50	IQR 40–60; range 20–60
Unplanned hospital admissions in last 6 months	Median 1	IQR 1–3; range 0–8
Female gender	10	
Lives alone	8	
Patient interviewed	17	
Interview setting		
In patients home^c^	13	
In hospital	5	
Carer interviewed		
Yes	7	
No (no carer nominated)	7	
No (carer declined)	4	
Carer characteristics	(*n* = 7)	
Relationship		
Spouse	3	
Son/daughter	4	
Lives with patient	5	
Female gender	5	
Interviewed separately	1	

a The CFS is scored from 0 to 9, with higher scores representing increasing frailty. Participants scoring 5 or more, corresponding to ‘Mildly frail: more evident slowing, requiring help with higher order activities of daily living’, were eligible for the study.

b The AKPS is scored from 0 to 100, with higher scores representing higher function. There was no cut off for AKPS. The highest score for participants was 60 = ‘able to care for most needs; but requires occasional assistance’.

c One participant was interviewed in supported accommodation.

The key influences on preferences, and their relation to response shift were as follows: (1) experiences of recent illness and care – the ‘*illness and care context*’ related to catalysts in response shift; (2) how participants’ coped with and responded to health changes, their ‘*adaptation to changes in health and care experiences*’, related to aspects of antecedents and mechanisms in response shift; (3) participants’ thoughts regarding the outcomes of their care, namely a desire to ‘*achieve normalit*y’, related to aspects of mechanisms in response shift and (4) social aspects, particularly the presence of family and friends, the ‘*social context*’, related to antecedents in response shift. These are further considered below (see supplementary material D for full coding frame).

### Illness and are context

The illness and care context formed the background to participants’ care preferences. Recent *care experien*ces were vividly described, and *changing health* was a universal concern among patients and carers. These two areas were sub-themes.

*Changing health* was a concern for all; in some cases, acute illness caused participants to change their preferences for future care. For example, loss of confidence following hospital admission might result in anxiety about being alone, which in turn might affect home care preferences:

Researcher (R): has … being back in hospital changed your priorities at all

Participant (P): Uh, it’s made me more afraid of being on my own at home. Whereas before I didn’t think too much about it and always thought oh um … you know you get all these scare tales but I’ll be alright I’ve always been alright before. But now this [collapse and hospitalisation] has happened I think ‘o crumbs this could happen again’ and I’m more frightened of being on my own, cause it doesn’t matter how many times you have a carer, when they go and shut that front door you’re on your own until the next time they come. So you know … yeah I suppose that’s made me think a bit differently about that. (89-year-old female, 1 recent hospital admission)

While most hoped for improvement, some felt they were in a cycle of illness and could not avoid hospitalisation. Recovery was infrequently considered by these participants, and the expression of preferences inhibited by a sense of helplessness:

P: I always when I come out of hospital I always say that that’s the last time and uh something happens and … I can’t fulfil that … because … well I’m back in hospital now. (70-year-old female, 8 recent hospital admissions)

This experience of illness was tiring and led to a sense of having ‘had enough’. This influenced preferences regarding prolongation versus quality of life:

P: Yeah, no, … with the … different – I got fed up, I keep going backwards and forwards, backwards and forwards and … you know, in uh different wards each time; I mean I think I’ve been at that blasted hospital more than they have [laughing]

R: Yeah. How does it make you feel?

P: I’d had enough. I thought and you know the girls said ‘You’ve been strong mum, you’ve fought this’ and I thought ‘you go and b***** yourself’ because I can’t fight no more. And that’s how I feel now. (79-year-old female, 3 recent hospital admissions)

*Care experiences* in hospital profoundly affected some participants, and while there were examples of high-quality care, this was especially the case when care was poor:

P: The feeling of utter helplessness and the feeling that nobody gives a damn but sorry (crying) that really got to me … I felt … why am I having to go through this [unrelieved pain] at this time of my life. (87-year-old female, 1 recent hospital admission)

Poor care experiences in hospital were highly upsetting and motivated participants to get out of hospital and avoid readmission:

P: It stops you getting better; it stops you, I wanted to get out of there. In fact I was so determined to get out of there that I pushed and pushed and pushed to get my release … and on the Monday having been in there 12 days when they came round I was determined or I go absolutely potty myself. (87-year-old female, 1 recent hospital admission)

Preferences for greater continuity of care were affected by experiences, especially at the point of discharge, where care was perceived as disjointed.

P: I always get a bit worried about that that when you go home, have the doctors in the hospital really contacted the others? (89-year-old female, 1 recent hospital admission)

### Adaptation to changes in health and care experiences

Participants’ responses included processes of *coping and adjustment*, changing *health awareness* and a range of *hopes and fears* about the future.

Coping styles varied greatly and affected how and what preferences would be expressed. Some participants coped by disengaging from what they considered a helpless situation, and hence preferred to avoid expression of preferences:

R: How would you feel if … if things didn’t improve.

P: Well there’s not much I can do about it. (75-year-old female, 1 recent hospital admission)

Carer (C): And then they got him into Pulmonary Rehab & he wasn’t able to do it and … you know. It’s just a progressive thing … just … do what you can. There’s no answer, no no one’s got an answer, no one.

R: And how does it make you feel knowing that?

C: We can’t do nothing about it. Cos we know there is no answer. We know that. So … we’ve got to live with it. Simple as that. (Carer (wife) of 82-year-old male, 1 recent hospital admission)

Others were more engaged, feeling that they had to keep going; this form of coping was associated with more active expression of preferences:

P: You think to yourself, no, you can’t give in, you can’t give in, but then I want to, so … I don’t know what makes me push myself. (79-year-old female, 3 recent hospital admissions)

Acute illness changed individuals’ *health awareness*, resulting in a changed outlook. This often resulted in more realistic preferences. Some referred to illness episodes as a ‘reality check’:

R: Having had this fall and being less well, do you think that’s changed your priorities and how you think about things?

P: … 4s … Well I suppose they have in a way, because I’m in a **different position** … 3s … so the priorities are basically to get home and move around the house. (84-year-old male, 1 recent hospital admission)

Most participants expressed *hopes* for recovery after acute illness. These hopes were not always realistic even when overall health awareness was good, suggesting that multiple awareness contexts existed concurrently. Whether realistic or not, hopes were an important motivator for people to engage with care:

P: I’ve got a wheelchair and I my husband can push me round if I need to but I hope in the future to be able to walk down … without this! (Indicates Zimmer). (85-year-old female, 0 recent hospital admissions)

### Achieving normality

Ultimately, most participants wanted to achieve a sense of normality in their daily lives. This was an overarching influence on preferences. Normality could be achieved either by ‘getting back to normal’ or, where this was not possible, by ‘finding a new normal’, and these were the two main sub-themes.

*Getting back to normal* was important to the extent that some could not consider anything else in relation to their preferences and would accept any care that got them closer to normality:

P: Because what you want from life is the normality … and um sometimes it’s very difficult to get that in hospital. (70-year-old female, 8 recent hospital admissions)

However, ‘normality’ was sometimes difficult to achieve, with new health problems intervening and requiring further adaptation. Nevertheless, the goal of ‘getting back to normal’ was an influence on many preferences and seen as a route to better quality of life:

P: Uh well because it would mean that you’re more mobile, which means you can do more, which means you’re a happier person I suppose, don’t have to ask for help so much; it just goes on and on doesn’t it. (89-year-old female, 1 recent hospital admission)

Some participants felt that they had achieved normality and focused on maintaining it:

C: And we will keep … doing what we do, won’t we?

P: oh yeah yeah it hasn’t stopped us doing anything. And it won’t do either, hopefully

C: you won’t just sit back and think ‘this is it’

P: no I won’t give up. … no as soon as you do that it is the beginning of the end. (82-year-old male and carer (wife), 2 recent hospital admissions)

Personal values were important as they influenced what participants considered normal was, and in turn whether they felt they had achieved it. Values varied, but a common thread was a desire to retain independence. Consequently, conflicts in preferences between wanting to be independent, versus accepting additional care were common. For one participant, independence manifested in a desire to spend time alone:

P: Well you know I’d like to get out, get out on my own, that’s what I want to do. You know [wife’s name] is with me all the time. (82-year-old male, 1 recent hospital admission)

#### Finding a new normal

Rather than trying to return to an ‘old’ normal, some participants reconceptualised what normality meant to them, seeking a new normal to fit with their situation. This could involve accepting health problems and integrating them into their narrative of day-to-day life:

P: I’ve had several falls, but that’s to me that’s a normal thing. (84-year-old male, 1 recent hospital admission)

P: **I don’t like it.** (laughter) Believe me I don’t like it but…[4s pause]… that’s where my life has gone so I’ve had to go with it. (85-year-old female, 0 recent hospital admissions)

When participants’ considered the future, they often thought of a situation where normality could no longer be maintained. This idea could be a grave concern, and participants recognised that their preferences might change:

P: [ominous, deep voice] Ohhhh yeahhh I think uh if it was to change I think well I think what I would have to do, is I’d have to uh I’d have to more or less stay in bed I suppose that would be that would be one solution. And uh you know to uh sort of get sort of meals on wheels and get your meals delivered that’s the only but I don’t want that. (93-year-old male, 2 recent hospital admissions)

While the prospect of deterioration distressed most participants, one divergent case, who was receiving palliative care, was matter of fact about future preferences:

P: She just wanted to know what my thoughts were if when the end comes do I want them to go through all the, you know, resuscitation and putting me on the life support and I said no, definitely not. (82-year-old female, 5 recent hospital admissions)

### Social context

The social context pervaded all aspects of participants’ preferences; in particular the people around an individual influenced their preferences. The influence of social support was most evident on preferences for place of care. This was a key difference for people living alone versus people living with someone. Having someone there added a sense of security:

R: How do you think it makes a difference having your … son around?

P: Well I always know he’s coming home … and he’s only in the next room if I need him during the night … (84-year-old male, 1 recent hospital admission)

Having someone there also promoted confidence, enabling participants to try things they wouldn’t do if alone, which was another way of getting closer to normality:

P: confidence I think in … whoever is with me. Cos I, I went up the stairs yesterday … and I think [physiotherapist] was with me … physio said don’t go up the stairs on your own, always have somebody. (80-year-old female, 3 recent hospital admissions)

Concerns of and about family influenced preferences, especially when family members were unwell. A sense of wanting to reduce burden on family members affected the care participants were willing to accept:

P: He does everything, the cooking, um the pick-up I’ll try not to keep making him come up the stairs, I’ll try to take the cup down cause I fancy another cup of tea and he’ll shout at me ‘what [did] you come down for? I’ve gotta come up’ and oh, and then you finish up arguing you know what I mean ‘I don’t want you to keep coming up here’, you know what I mean; ‘your knees are getting worse’ which they are. (79-year-old female, 3 recent hospital admissions)

This occasionally resulted in disagreements, for example, when family members wanted to provide care, but participants felt differently due to fears of overburdening them. Financial considerations were sometimes important, influencing place of care options particularly when participants considered moving to a care home, or receiving private care:

P: I might have to go into a care home anyway I don’t know. See how things … work out.

R: And how do you feel about that prospect?

P: (3s pause) … well … (3s pause) … I think I could afford to go into a private one rather than NHS. (84-year-old male, 2 recent hospital admissions)

**Figure 1.**
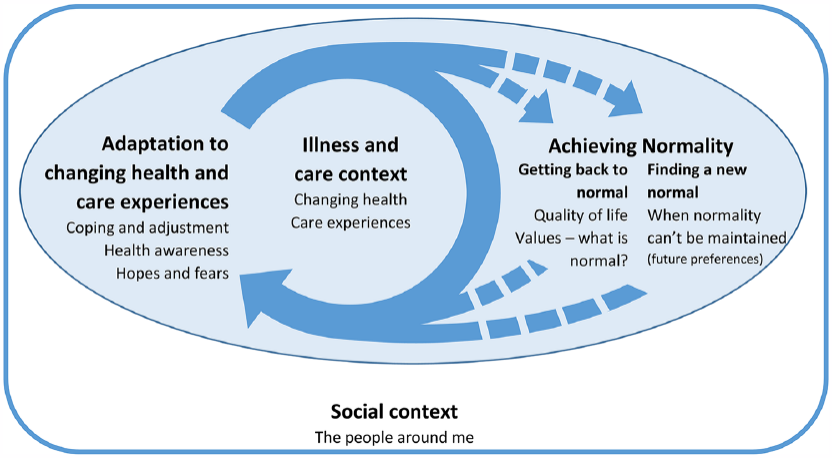
Thematic model of influences on care preferences following acute illness. Please note that all aspects of this model relate to influences on care preferences; hence, preferences themselves are not specified within the model.

We produced a model of the influences on preferences in this population (Figure 1). In this model, the illness and care context sit centrally. Participants adapt to changing health and care experiences, with the ultimate goal of achieving normality. Most want to ‘get back to normal, but where this isn’t possible, participants may seek a ‘new normal’. The dashed arrows indicate that sometimes normality is unachievable due to new health changes or care experiences, which require further adaptation. The social context is important throughout.

## Discussion

### Main findings

We found that in the context of recent acute illness, participants focused on the outcomes they wished to achieve from their care; hence, preferences were influenced by a desire to remain independent and ‘get back to normal’. However, the unpredictable nature of illness sometimes meant that this was unachievable, and some participants sought a ‘new normal’. Participants experienced a cycle of illness and recovery, with preferences influenced by illness and care experiences, and by adaptation to ongoing health changes. The social context, particularly support from family and friends, influenced preferences throughout.

### What this study adds

The concept of normality in the context of illness is not a new one. Biographical disruption theory considers illness as a break in life narrative – a loss of normality.^38,39^ Regaining a narrative – getting back to normal – is an important way to cope with illness. Acute illness can be regarded as a crisis to be gotten past,^28^ and getting back on track after a crisis is another way of describing normality. What differs in this population is that frailty limits functional recovery, and recovery is less certain.^26^ This means that rather than a crisis to be ‘got over’, acute illness may represent the point when normality is lost and cannot be regained. Getting back to normal then becomes unachievable, and sometimes individuals must balance losses and continuity to find a new normal.^40,41^

Response shift theory may explain how normality influences preferences, and why some participants sought a new normal, rather than trying to regain an old normal. Participants may either reprioritise what is important to them, or reconceptualise what normal means.^22^ This resonates with Calman’s hypothesis that quality of life is the gap between actual experience and expectation.^42^ One can narrow this gap by improving health status (i.e. getting back to normal). However, the gap can also be narrowed (and quality of life improved) by moderating expectations (i.e. finding a new normal). Those who reconceptualise normality may consequently change their preferences in line with their new normal.

### Implications for practice

Although care quality and specific care experiences did influence preferences, most participants focused on desired outcomes after acute illness,^43^ that is, whatever would help them achieve normality,^44^ or as Sandsdalen put it, ‘live a meaningful life’.^45^ This has important clinical implications, especially for person-centred discharge planning, and advance care planning.^46^ For discharge planning, rather than discuss care post-discharge in terms of its processes (number of daily visits, whom will be involved), it makes more sense to focus initially on goals of care,^11^ by discussing what is an achievable normal in the context of what matters to an individual, and then discuss care in relation to this. To determine what normal means for someone, we need insight into the person and their circumstances,^47^ including ‘where’ they are in the cycle of illness. If the ‘normal’ that people aspire to is understood, clinicians can suggest care which may enable people to maintain this identity and the dignity in their daily life.^48^ Or where normality cannot be maintained, clinicians may be able to help people find a new achievable normal. Exploring appropriate questions to develop this understanding is a target for future research, as has been considered to some extent for advance care planning.^49^ Given the importance of social context, clinicians should also involve families in conversations about preferences, and consider patients and their families together as a unit of care. They should also consider how the illness context, and different ways of coping may inhibit or enable the expression of preferences.

Our findings also have implications for advance care planning, and support an iterative approach to planning.^50^ By understanding what may influence preferences, more flexible advanced care plans may be possible, which accommodate the unpredictable nature of advanced illness. Achieving normality may be a useful concept for advance care planning, and what an individual considers as an acceptable normal may be something that could guide future decision makers.

We still know little about how preferences are influenced over time and through different stages of illness. Prospective longitudinal research is needed to describe the stability and influences on preference over time. Future research should explore in greater depth the concepts of getting back to normal, and finding a new normal, in order to better understand why, when and how this shift may occur.

### Strengths/limitations

This study collected rich data from a ‘difficult to reach’ population.^51^ Theoretical sampling enabled access to diverse experiences.^34^ Our topic-guide was developed iteratively, with input from PPI representatives, who also contributed to interpretation, optimising the relevance of results to the population of interest. However, a single interview may have been insufficient to fully capture experiences in this sensitive area, and all but one participating caregivers were interviewed jointly, potentially inhibiting frank discussion of preferences. A single researcher undertook all interviews, which may have led to interpretation bias. This risk was mitigated using a reflexive diary, discussing findings within the research team, and by double-coding a random subset of transcripts. While we filled a maximum-variation sampling frame, our findings are not necessarily transferrable to other populations, for example, those of diverse ethnicity, from rural settings, nursing home residents, or those receiving specialist palliative care. In addition, we focused mainly on acute illness severe enough to cause hospitalisation; acute illness is often managed in primary care and this may influence preferences differently.

## Conclusion

Getting back to normal or finding a new normal in the face of health changes are central to the development of care preferences in frail older people with recent acute illness. By exploring what is an achievable normal for their patients, clinicians may be better able to plan and deliver care that takes preferences into account. Influences on the stability of preferences remain an important evidence gap.

## Supplemental Material

Supplementary_material – Supplemental material for Finding a ‘new normal’ following acute illness: A qualitative study of influences on frail older people’s care preferences Click here for additional data file. Supplemental material, Supplementary_material for Finding a ‘new normal’ following acute illness: A qualitative study of influences on frail older people’s care preferences by Simon Noah Etkind, Natasha Lovell, Caroline Jane Nicholson, Irene J Higginson and Fliss EM Murtagh in Palliative Medicine
