# Supplementary material for: Finding a ‘new normal’ following acute illness: A qualitative study of influences on frail older people’s care preferences
Source: Palliat Med. 2018 Dec 7;33(3):301–11. doi: 10.1177/0269216318817706 (PMC6376597; doi:10.1177/0269216318817706)
Supplement: Supplementary_material – Supplemental material for Finding a ‘new normal’ following acute illness: A qualitative study of influences on frail older people’s care preferences [file Supplementary_material.docx]

**Supplementary Material A: Qualitative sampling frame**

| **Criterion** | **Values** | |
| --- | --- | --- |
| Hospital Admissions | **≤1 admission in last 6 months**  10  (8 – 10) | **>1 admission in last 6 months**  8  (8 – 10) |
| Level of Function | **Karnofsky performance status ≥60**  7  (8 – 10) | **Karnofsky performance status ≤50**  11  (8 – 10) |
| Living Status | **Lives with someone**  10  (8 – 10) | **Lives alone**  8  (8 – 10) |
| Age | **Age <85**  9  (8 – 10) | **Age ≥ 85**  9  (8 – 10) |

Note: The numbers in the sampling frame refer to number of participants. The target number of participants for each part of the coding frame is denoted in brackets. The actual number sampled is in red

**Supplementary Material B: Full topic guides**

Topic guide notes for researchers.

1. How to use this document: The topic guide is not designed to be read out to participants word for word. Rather it is a guide to the topic areas that are important for this research. The questions on the left hand side are suggestions for open questions to get into each topic area, with more specific areas that *might* be explored on the right. Not all questions need to be asked in every interview, and some topic areas will be covered during open conversation.

1. Joint interviews: Some topics may be difficult for patients to express in front of their family/caregivers and vice versa. Because of this, all interviews will be conducted separately where this is possible and acceptable to the participants. However, if patients & family/carers request to be interviewed together, this will be respected. In this situation, an opportunity for either or both to talk separately at the end will be offered. For joint interviews, the patient topic guide should be followed, but opportunity given to the carer to express their views on the issues raised.

*Patient topic guide*

*Thank you for taking part in this interview. Through this interview we hope to understand more about your experience of living with illness, how you feel about the care you have received and things that are particularly important to you*.

| 1. **Experience of illness –** *first I’d like to ask you about your health* |
| --- |

| Topic area | Potential areas to be explored |
| --- | --- |
| Can you tell me a bit about how your health has been over the last 3 months? | Different illnesses, understanding of illness & of care received.  Is health improving, staying the same or worsening? |
| Are you limited by health problems? How do health problems limit you day to day? | Effects on day to day life, isolation, maintaining independence, social support, external help |
| What are your main health concerns at the moment? | Explore concerns, type, and number, is anything else concerning you? What’s the worst thing? Explore symptoms & how they affect quality of life. Explore psychological symptoms |
| What helps you cope with your illness? Is anything especially hard to cope with? | Support mechanisms, gaps in support, family support. |

| **2. Experience of healthcare & accessing care** *– now I’d like to ask you about your*  *experiences of healthcare services.* |
| --- |

| Topic area | Potential areas to be explored |
| --- | --- |
| Could you tell me about the care you have received for your health in the last 3 months? | Types of care received, volume of service use, care burden. Explore kinds of care e.g. GP, district nursing. Explore how patient came to receive these kinds of care – how/why did it happen? Explore what patient has found helpful/unhelpful, |
| Have you been in hospital recently? Please tell me about your experience in hospital. | Reason for admission, experiences during admission, especially focus on the run up to hospital admission |
| What changes would you like to make to the healthcare you’ve received? What would make it better | Explore models of care and types of services that patients would prefer. What makes a service more acceptable? What 1 change would make the biggest difference? Explore aspects relevant to palliative care |
| How easy is it for you to see your GP/others involved in your care? | Accessibility of services, opening times, distance away, transport, practical issues affecting ability to reach services. |
| Do you feel your GP, hospital doctor & nurses & other staff are working well together? | Care co-ordination |
| What has made it easier or harder to get the care that you have wanted? | Facilitators and barriers to care. |
| Do you ever miss appointments? | Why does this happen? What might stop this |

| **3. Preferences** *– the next section is about what’s important to you in your life, and also what is important with regard to your health* |
| --- |

| Topic area | Potential areas to be explored |
| --- | --- |
| What would you say are the most important things/things you value most in your life at the moment? | Priorities – not necessarily health related. Explore how easy it is for patient sustain/achieve important things?  Anything they want to achieve – life goals. How are these affected by health? |
| And in terms of your health: what is important to you with regards to your health and the care you receive? | Preferences for type of care, aggressiveness of care, level of input from professionals  Level of engagement with illness.  How do you decide what is important? Healthcare goals |
| How do you decide what’s important for your health? | Explore how preferences and priorities develop. What things are taken into account. Who is involved  How do people prioritise |
| Do you think what is important changes over time? | Explore ideas of how preferences may change over time. Do different things become important at different times? What changes what priorities |
| How do you make choices about your health? When you have to make choices how much information do you prefer to have | Decision making preferences. |

| **4. Ideas about the future** *– finally I’d like to ask about the future* |
| --- |

| Topic area | Potential areas to be explored |
| --- | --- |
| How do you see your health changing in the near future/further ahead? | Explore concerns and thoughts about the future, ideas about what might happen |
| Are you someone who tends to think about what the future may hold for your health? | Temporal focus – does patient consider their future, do they plan ahead? If yes, what do they think about the future? If not, why not? |
| If your health were to worsen, are there treatments that you would or wouldn’t want to receive? Or things that you would/wouldn’t want to happen to you? | Explore preferences for future care  If appropriate, explore perceptions of palliative care |

*Thank you, your answers have been really helpful. We have now come to the end of the interview. Is there anything else you would like to share before we finish?* [END OF INTERVIEW]

*Carer topic guide*

*Thank you for agreeing to participate in this interview. Through this interview we hope to understand more about <patient’s> illness and how it has affected you both.*

| 1. **Experience of illness & Preferences –** *First I’d like to ask a bit about yourself and about <patient>* |
| --- |

| Open Questions | Potential areas to be explored |
| --- | --- |
| How are you related to <patient>? How is your health? |  |
| Could you describe what has been happening with <patients> health in the last 3 months? | Carers view on patient illness |
| How has <patient’s> illness affected you? | Carer burden, physical & mental difficulties, Isolation, positive aspects of care. What is hard/easy |
| Is there anyone who provides you with support? | Who provides support? What kind of support, how has this helped? If not, who might provide support? |
| How do you cope when things are hard? | Help seeking, access to support, unmet need |
| Who can you ask for help if needed urgently? | Point of contact, how was this made known? |
| What is important to you and <patient> to help you live with good quality of life? | Preferences for care & priorities for quality of life |

| **2. Experience of care/Access** *– now I’d like to ask you a bit about <patients> care* |
| --- |

| Open Questions | Potential areas to be explored |
| --- | --- |
| Can you tell me about <patient’s> care in the last 3 months? | Care experience of healthcare. Good/bad aspects. |
| How would you describe your experience of the hospital/a&e system? | Carer perspective on acute healthcare usage. Experience of care, |
| Has <patient> been able to get the kind of care they need, when they need it? | Facilitators/barriers. Care co-ordination  What has made this more difficult/easier? |
| How easy is it for you to see <patient’s> GP/others involved in <patient’s> care? |  |
|  |  |

| **3. Ideas about the future** *– finally I’d like to ask you about how things may change in the future* |
| --- |

| Open Questions | Potential areas to be explored |
| --- | --- |
| How do you see <patient’s> health changing over the coming months? | Explore thoughts and concerns about the future, what do they expect/not expect to happen. |
| If <patient> were to become less well in future, what would be important to make sure they were looked after as well as possible. | Explore elements relevant to palliative care. Anything they wouldn’t want to happen? |

*Thank you, your answers have been really helpful. We have now come to the end of the interview. Is there anything else you would like to share before we finish?* [END OF INTERVIEW]

**Supplementary Material C. Additional details and reflection on analysis and interpretation:**

The analysis for this study followed the principles of thematic qualitative analysis, and proceeded according to the following process. Analysis included inductive and deductive components, and utilised two frameworks: Response Shift theory (supplementary figure 1), and the Etkind et al ecological systems-based model of influences on care preferences (supplementary figure 2).

Stages of analysis were as follows:

- **Recordings transcribed** verbatim, paying attention to non-verbal cues, emphasis and pauses.
- **Transcripts read and re-read** to familiarise with data.
- **Transcripts coded inductively** for data relevant to influences on preferences. No formal reference was made to theoretical frameworks at this stage, but the coding authors were aware of the frameworks in question, particularly the individual-illness-family and care context domains of the Etkind et al model.
- **Double coding** of a random subset of 3 transcripts to assess the initial coding frame.
- **Grouping of related codes into themes and sub themes.** At this stage the emergent themes were interpreted in the light of existing theories and frameworks as follows:
  - Response shift theory was used to explore the processes by which preferences might develop and be influenced. We considered our data in terms of the stages of response shift (supplementary figure 1). The data did not fit perfectly into response shift, and no attempt was made to make the data fit. However considerable similarity was noted in some areas, particularly:
    - Coping and social comparisons as ways in which people adapted to changing health
    - Changing health and care experience as catalyst
    - Achieving normality and Goal reordering/reconceptualization/reprioritisation response shift
  - The fit with the Etkind et al model of influences on preferences was also explored, primarily as a way of assessing whether we were missing known influences on preferences in older people, and whether there was anything in our data that hadn’t been recorded previously. Key similarities were:
    - The importance of social context and presence/views of family and loved ones as an influence
    - Current illness concerns and care experiences.
    - The role of expectations, and changes in expectations
  - There were some differences:
    - Sociodemographic influences not evident in our data – this wasn’t something that we expected to identify using qualitative methodology
    - The cyclical nature of acute illness wasn’t evident in the systematic review, but this likely represents the difference in population between this study and the systematic review.
    - Achieving normality was more prominent in this qualitative study, though some of the sub themes – e.g. underlying values – were evident as influences in the systematic review model.
- **Discussion and interpretation of thematic model** with research team and PPI representatives. The research team were familiar with the data, and discussed the thematic framework.

PPI representatives were shown key aspects of the data to enable familiarisation and were then asked for their interpretations. They were then shown the full thematic model and asked to comment on how well this fit with their interpretations. The thematic model was refined based on these discussions, in particular, the sub-themes of the “Achieving normality” theme were refined and more carefully distinguished in the final model.


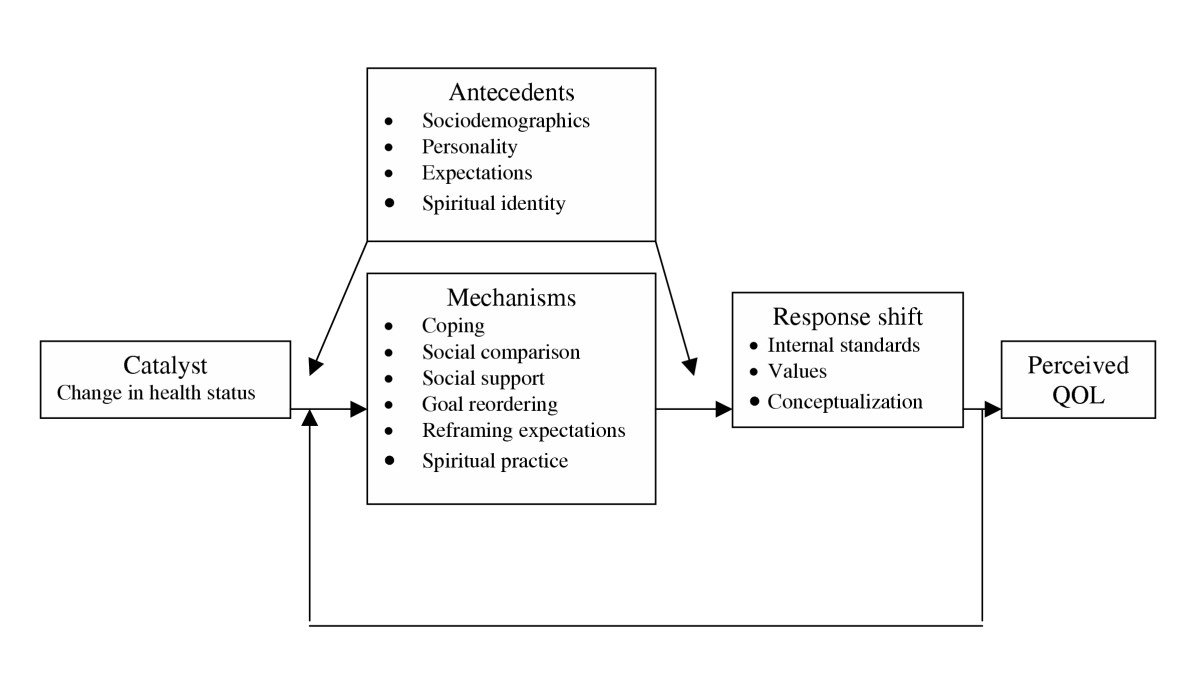
*Supplementary figure 1.* Response shift. From Schwartz and Sprangers, 2000

**Reference:** Schwartz CE, Sprangers MA: **Adaptation to changing health: Response shift in quality-of-life research.** Washington D.C, USA: American Psychological Association; 2000.

*Supplementary figure 2.* Model of influences on preferences from Etkind et al 2018.

*
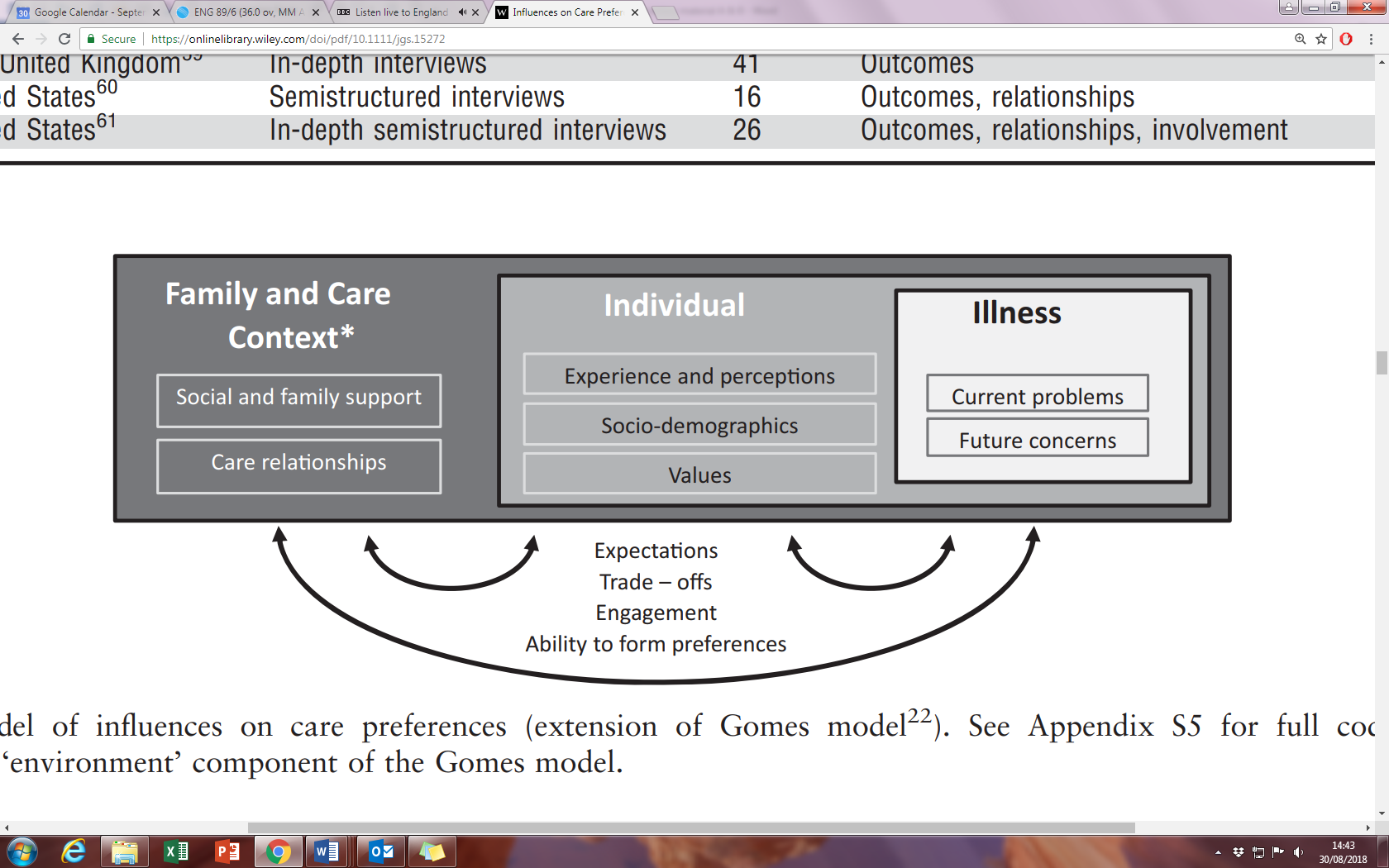
*

**Reference:** Etkind SN, Bone AE, Lovell N, Murtagh FE, Higginson IJ: **Influences on care preferences of older people with advanced illness: A systematic review and thematic synthesis.** *JAGS* 2018, **66:**1031-1039.

**Supplementary material D. Detailed Coding Frame**

NB. Main themes are highlighted in larger font, with sub themes in bold. Below this, nodes contained within each sub-theme are described

| **Themes, sub-themes and nodes.** | **Description** |
| --- | --- |
| Illness & care contexts | Changing health, and new and ongoing care experiences, form the immediate contexts to how preferences are influenced. These acute contexts e.g. a fall, acute illness or new care experience are the backdrop to how preferences are influenced |
| **Changing health** | The impact and influence of illness. Often change is the key attribute of health problems, as reflected in the theme name |
| Acute Illness | Acute illness was an important event for all participants given the population, and these experiences and their effect are described |
| Calling for help | When participants felt unable to manage and sought help or advice |
| Confusion & disorientation | Cognitive changes and confusion associated with acute illness, and the consequences of this on preferences |
| Falls | Falls are a key precipitator of acute illness in this group and are much feared. The impact of falls may be immense, and may precipitate a reassessment of situation and a change in preferences. |
| Illness Trajectories | Descriptions of different illness trajectories as experienced by participants |
| Deterioration | When people feel they are getting worse |
| Recovery | When people feel they are improving, including time taken to recover. |
| Uncertainty | Uncertain illness trajectories, including descriptions of a vicious cycle of illness. |
| Impact of illness | How illness limits someone, how it affects their life |
| Function | The importance of changes in function & the influence of this on participants’ preferences. |
| Priorities related to level of function | The importance of level of function as a determinant of preferences. Participants often referred to changes in function as impacting on their preferences. “ if I could do x, then I’d like y”. |
| Losses | This node describes the losses people experience with acute and chronic illness. Includes loss of function, things they can no longer do and the impact of these losses on their lives. In particular loss of **confidence** is a key part of this code, as is the impact of missing out on important events. |
| Physical health problems | The physical health problems faced by participants, in particular new problems, long term health problems and multimorbidity. Competing health priorities are a feature of physical illness in this group and require a response in terms of preferences. Also includes the treatments and tests participants must undergo. 'feeling ill' is a sub-code, focusing on how physical health problems feel to participants |
| Feeling ill | How people feel physically in relation to health problems, in particular a focus on symptoms and pain. |
| Psychological impact of illness | Illness may result in a number of adverse feelings - several fears (of deterioration, dying, being alone), feelings of **helplessness**, loneliness, and low mood are common and affect preferences. Stigma is apparent and some people feel judged, especially in the context of mental illness. Physical and psychological impacts often run together, and the two cannot be seen as separate. |
| **Experiences of care** | Descriptions of experiences of care, both acute and long term. |
| Care context | Experiences of care in different settings and how this influences preferences |
| Care at home | Experiences of care at home, including process of being discharged, accessing social and private care, and how these experiences may influence ongoing preferences. Also includes the importance of being at home because "home is home". |
| Experience of hospital | Descriptions of hospital experiences. In particular the experience of hospitals at night, hospital food, seeing other patients (especially those unwell with dementia) and the effects of hospital experiences on preferences. |
| Hospice | Experiences of hospice care, including the extra support offered |
| Care relationships | Relationships with different health professionals, and how these affect preferences. Includes the importance of **continuity**, and communication |
| Communication | The impact of different communication experiences with health professionals, and individuals preferences for communication at different stages. |
| Continuity | When participants consider continuity of care, the benefits this may bring and the effects if care continuity is lost. Based on their expectations regarding care continuity, participants may amend their preferences. |
| Quality of care | Experiences of aspects of care relating to its quality. |
| Access to & variability of care | Access problems, changes or variability of care |
| Bad experiences of care | When care doesn’t meet expectations or there are problems. Particularly if people aren’t treated as they would like to be, where there are conflicts in care, or *where care isn't delivered in line with preferences*. |
| Good experience of care | An experience of care that is satisfactory or better. Especially care that is responsive, experience of staff making an effort to help, and whether this influences preferences. |
| Adaptation to changing health and care experiences. | Ways in which people adapt to health changes and cope with new limitations. In particular coping with changed level of function and how this impacts on preferences. |
| **Coping and Adjusting** | The ways in which people cope with their changing health/ care experiences and come to terms with them. This fits with other recognised models of coping such as response shift. Coping is a dynamic process - and may involve disengagement & not expressing preferences. Equally, resilience, persistence and social comparisons may help people to come to terms with changes. |
| Battling through and keeping going. | An approach to coping which involves keeping going through problems. Often linked with a desire to be independent |
| Disengagement | When participants express disengagement form their illness, either by expressing that there is nothing to be done, that they have lost control, or that uncertainty precludes planning. Participants may also not want to think ahead. This form of coping results in people not expressing preferences. Linked to a feeling of helplessness. |
| Motivation | Things that motivated participants to recover, or the sense of motivation they felt. Linked to preferences about care outcomes, and achieving normality |
| Putting suffering in context | When people make comparisons to put their suffering in context, and how this affects preferences. Comparisons might be to past or expected future health states, to hypothetical 'worse' states, or social comparisons to others' health. |
| Self-confidence. resilience | Some participants expressed confidence in themselves or felt resilient. They were able to ‘get through’ acute illness. Often linked to independence |
| Spiritual coping | Participants occasionally referred to spiritual reasons which enabled them to keep going. Eg ‘god has sent me back for a reason |
| Trade-offs in preferences | Often two preferences, e.g. a desire to remain independent, and wanting to have someone around conflicted. Participants traded off one thing against the other |
| World shrinking | Linked to the experience of losses, participants often felt that they were able to do less and that their world was shrinking. They had to adjust to this new situation, and this might necessitate a change in preferences |
| **Health awareness** | The impact of health awareness on preferences. Participants might consider that their health is 'normal for their age' and therefore not expect any special care. They may have varying awareness of how much support they need. For some, other factors such as hospitalisation may be a ‘reality check’ increasing awareness and changing preferences. Hopes and expectations are a sub-theme |
| **Hopes and Expectations** | When people express hopes and expectations about their health. These depend on their health awareness and coping strategies, and may have varying levels of realism. Some expectations may be negative "how long have I got left?” This links to the psychological impact of illness. Changing expectations is a key driver of changing preferences. |
|  |  |
| Achieving Normality | This theme describes one key influence of preferences following acute illness - the desire to achieve normality. This fits with the Narrative Disruption and Crisis theories of acute illness. People's preferences are driven by desired care outcomes relating to normality - whether that means being pain free, being in one's usual environment, or being able to do day to day things. |
| **Getting back to normal** | The influence on preferences of a desire to 'get back to normal', or to maintain a sense of normality or narrative continuity |
| Quality of life | Expressions relating to achieving or maintaining a normal quality of life. Quality of life may be expressed in terms of achieving happiness, living day to day, or focusing in place of care. |
| Focusing on place of care | When participants focus on place of care preferences in order to achieve quality of life. This includes expressions of why place of care is important including the sense of why home is an important place to be, and what home means to people. |
| Living day to day | Most participants expressed an element of wanting to live day to day, doing their usual activities, or focusing on getting out and doing things. This day to day focus was more common than a focus on the future and on illness progression, and may be driven by the uncertainty of prognosis. Again, living day to day was an inhibitor of expressing preferences for the future, and could be linked to dis-engaged coping. |
| Values - what is normal | Values regarding what normal is. If people wish to maintain or achieve normality, then values regarding what is normal may influence preferences by influencing what someone wishes to achieve. Values may be long held, or may be based on life experiences. A key value in this population is independence, which is given its own sub-code. |
| Independence | When participants express independence. This is often a key underlying driver of care preferences. Specific areas that may be important are control, particularly controlling what external help is provided, and being able to be alone. Independence is a core value, and a long term driver of preferences |
| **Finding a new normal** | Finding a new normal is a closely related but distinct theme to getting back to normal. It is an influence on preferences, but also a response to the situation when normality cannot be maintained. Reconceptualising what normal means enables participants to find a new normal. If the concept of normal changes, then care preferences may also change. |
| When normality can no longer be maintained - future preferences | Some participants think ahead to what their preferences would be in future. This may influence current place of care, communication or other preferences. Whilst some preferences for future focus on maintaining normality, there is acknowledgement that normality will stop, and so end of life preferences are then expressed. Relates to health awareness |
| Social Context | The wider social environment influences preferences as it affects what participants think can be possible. Having people around is hugely important, especially as an influence on place of care preferences. |
| **The people around me** | Family, friends and neighbours – the influence of having people around. Includes the influence of **isolation** or not having people around. |
| Family | The importance and influence of family members, either co-habitants, or those living further afield. |
| Concerns of and about family | When participants are concerned about their family, not wanting to be a burden. This is often a key priority which changes other preferences.  Includes perceived concerns of family members, and conflicts between family concerns and patient views. |
| Impact on family & carer concerns | Descriptions of the impact of illness on family carers, including when family are delivering care.  Also includes specific concerns and worries expressed by carers, and how these might influence preferences. |
| Caring for others | When participants described their own caring responsibilities, including looking after unwell relatives. Includes previous caring responsibilities and how these may influence current personal preferences, and how the death of loved ones may influence preferences. |
| Friends, neighbours and local people | The influence of non-family interactions, whether social interactions, the importance of good friends; or negative interactions including discrimination |
| The importance of having someone there | The importance of having people around. E.g. evidence of when the presence of family influences preferences, evidence of preferences being a joint consideration, the importance of feeling accepted and understood. Includes the impact of not having someone there – I.e. social isolation |
| **Financial** | Financial issues, including ability to pay for care at home, or to afford a private care home, were an influence on future place of care preferences especially in those who were better off. |

**Supplementary material E**: GRIPP2 short form reporting of patient and public involvement in this study.

| **Section and topic** | **Item** | **Reported on page No** | **Additional Detail** |
| --- | --- | --- | --- |
| 1: Aim | Report the aim of PPI in the study | Methods page 9 | We included PPI in this study to ensure our exploration of preferences remained relevant to the population of interest, and to avoid making interpretations older people’s preferences without including them in the interpretation process. We also wanted to ensure the study materials were clear to facilitate participation. |
| 2: Methods | Provide a clear description of the methods used for PPI in the study | Methods page 9 | We set up a project advisory group at the start of the study (prior to ethical approval). The group, made up of 4 representatives, met face to face on a 6 monthly basis, and was also involved in email correspondence in between. Members were given information about the study, and were asked to review the study materials. Findings were presented to them and interpretation was discussed. |
| 3: Study results | Outcomes—Report the results of PPI in the study, including both positive and negative outcomes | Discussion page 22 | PPI resulted in clear documentation which was well received during ethical approval, and by participants.  Input at the interpretation phase was invaluable to sense-check our results and ensure relevance. |
| 4: Discussion and conclusions | Outcomes—Comment on the extent to which PPI influenced the study overall. Describe positive and negative effects | Discussion page 22 | Improved relevance of results, and potential benefits in the recruitment process were seen. |
| 5: Reflections/ critical perspective | Comment critically on the study, reflecting on the things that went well and those that did not, so others can learn from this experience | This table | Involvement at the interpretation stage was of great value. Additional involvement in coding and analysis would have enabled PPI representatives to gain a more in-depth understanding of the data, but this wasn’t feasible in this instance. Whilst review of study documents was useful, the resultant study information sheet was still too long. In future, a clearer conversation about the role and scope of PPI at the beginning of the study may result in further improvements. |

**Reference:** Staniszewska S, Brett J, Simera I, Seers K, Mockford C, Goodlad S, et al. GRIPP2 reporting checklists: tools to improve reporting of patient and public involvement in research. Research involvement and engagement. 2017;3(1):13.
